# High end-of-life incidence of seizures and status epilepticus in patients with primary and secondary brain tumors

**DOI:** 10.1007/s11060-022-04133-1

**Published:** 2022-11-03

**Authors:** Sophie von Brauchitsch, Adam Strzelczyk, Felix Rosenow, Elisabeth Neuhaus, Daniel Dubinski, Joachim P. Steinbach, Martin Voss

**Affiliations:** 1 Epilepsy Center Frankfurt Rhine-Main, Department of Neurology, University Hospital/ Goethe University Frankfurt, Frankfurt am Main, Germany; 2 LOEWE Center for Personalized and Translational Epilepsy Research (CePTER), Goethe University Frankfurt, Frankfurt am Main, Germany; 3 Institute of Neuroradiology, University Hospital/ Goethe University Frankfurt, Frankfurt am Main, Germany; 4 Department of Neurosurgery, Faculty of Medicine, University of Rostock, Rostock, Germany; 5 Dr. Senckenberg Institute of Neurooncology, University Hospital/ Goethe University Frankfurt, Frankfurt am Main, Germany; 6 University Cancer Center Frankfurt (UCT), University Hospital/Goethe University Frankfurt, Frankfurt am Main, Germany; 7 German Cancer Consortium (DKTK), Partner Site Frankfurt/Mainz, and German Cancer Research Center (DKFZ), Heidelberg, Germany

**Keywords:** End-of-life, Brain tumor, Epileptic seizures, Status epilepticus, EEG

## Abstract

**Purpose:**

Seizures pose a significant burden in patients with primary and secondary brain tumors during the end-of-life period. A wide range of 6 to 56% of clinically observed epileptic seizures at the end of life has been reported. We aimed to analyse the incidence of epileptic seizures at the end of life in brain tumor patients more accurately using not only clinical but also electrophysiological findings.

**Methods:**

This retrospective, single center study included brain tumor patients who died during the stay on the ward or within 7 days after discharge between 01/2015 and 08/2020. Clinical observation of seizures derived from the original medical records and EEG findings (within 45 days prior to death) were analyzed to determine the incidence of seizures in that period.

**Results:**

Of the 68 eligible patients, 50 patients (73.5%) suffered from seizures within 45 days prior to death, of which n = 24 had a status epilepticus. The diagnosis of seizures/ status epilepticus was determined either by the presentation of clinical signs in 45 patients and if not, by the detection of a (possible) non-convulsive status epilepticus in the EEG of five patients.

**Conclusion:**

In the presence of neurologically trained staff and with the frequent use of routine EEG, we were able to identify seizures and to distinguish status epilepticus from encephalopathy/ hypoactive delirium. We detected a higher incidence of seizures and status epilepticus at the end of life in neurooncological patients than previously reported.

## Introduction

A common symptom of primary brain tumors and brain metastases are seizures. In 30–50% of the patients, it is the first symptom leading to the initial diagnosis [1]. The risk of developing an epilepsy in patients with primary brain tumor is between 20 and 80% and exceeds the risk for patients with brain metastases [2].

The exact numbers of patients suffering from seizures during the course of the disease might be higher than reported or clinically estimated. In a previous study, 2% (n = 24) of 1101 brain tumor patients in the emergency department (of those 259 were randomly EEG-screened) presented with a non-convulsive status epilepticus (NCSE) with 79% of the 24 patients with NCSE having subclinical seizures [3]. These data indicate that NCSE are rather challenging to diagnose and often not adequately treated. Seizures also present a great burden for patients and relatives in the end-of-life phase [2]. “End of life” is usually defined as the period once the specific anti-tumor treatment is evaluated as futile [4]. The duration is between days to months for the individual patient. Incidence of clinical observed seizures at the end of life (ranging 3 to 300 days) in retrospective studies has been reported in a wide range of 6 to 56% [4].

Previous studies concerning seizures at the end of life in neuro-oncological patients are mostly set on palliative care units, hospices [5–7], and outpatient/home care [8, 9] and based on telephone surveys, questionnaires and history taking during regular home visits [10–12]. Furthermore, the studies involving inpatients considered the entirety of symptoms at the end of life with seizures being not the major focus [4, 13]. In summary, based on the circumstances of the previous studies, we hypothesized that the incidence of epileptic seizures for patients at the end of life exceed the previously reported rate.

We therefore performed retrospective study examining brain tumor patients within the last 45 days prior to death using the advantage of clinical observation by specifically trained neurologists and nursing staff on our neuro-oncological/ epileptologic hybrid ward as well as the access of routine EEG on a low threshold.

## Method

The retrospective, single center study was performed at the department of neurology of the University Hospital Frankfurt. Data were derived from the original medical records (including physician’s letters, documentation of ward rounds, documentation of special events, drug charts, reports of diagnostic findings from the department of (neuro-)pathology, neuroradiology and epileptology).

**Fig. 1:**
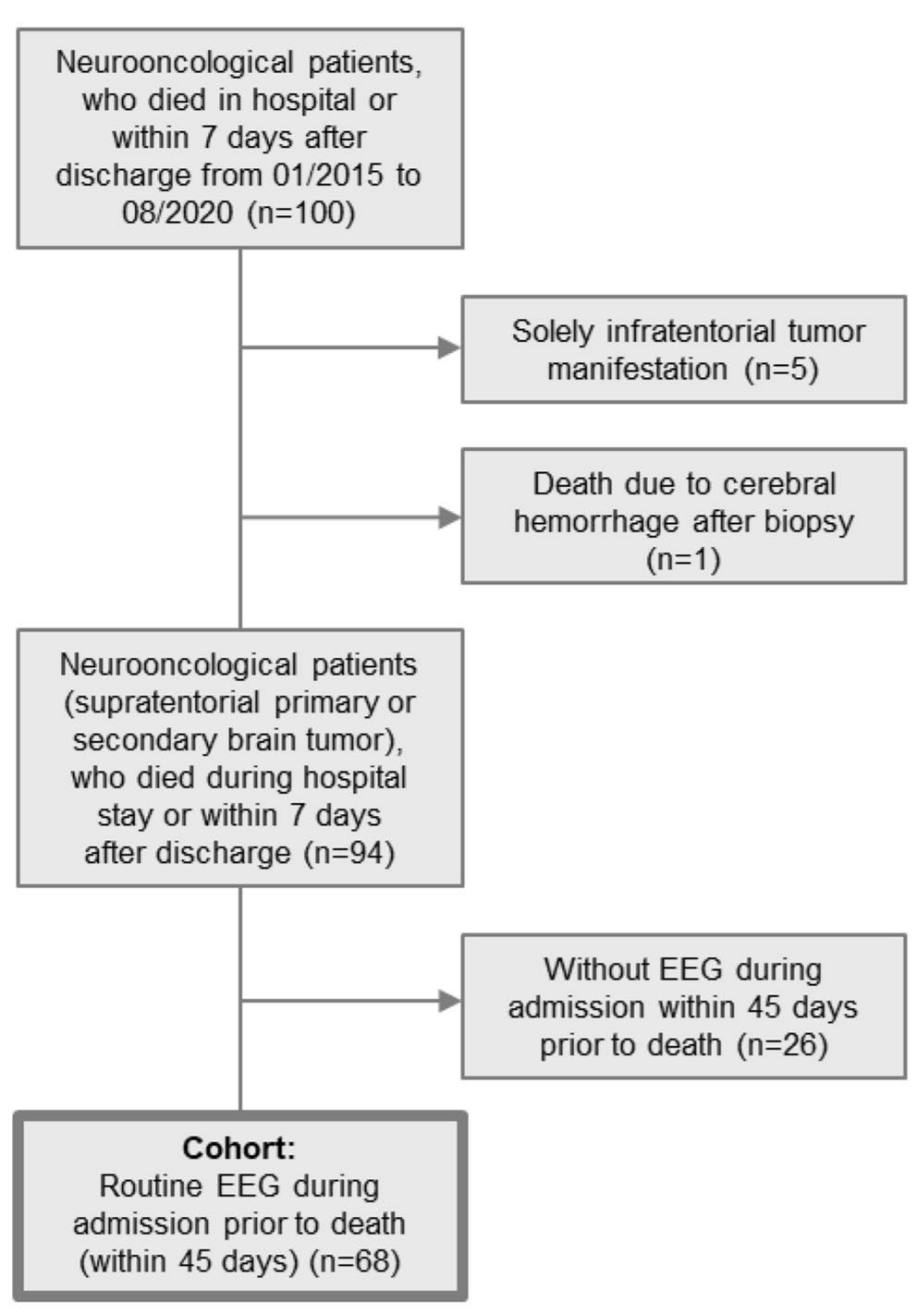
Consort diagram. All neurooncological patients who died while admitted in hospital or within 7 days after discharge were considered. Patients with solely infratentorial tumor manifestations were excluded. Of those left, only patients who received an EEG within 45 days prior to death were included.

We extracted a list of all neuro-oncological patients who had been admitted to the neurological wards during the period from January 1st 2015 and August 31st 2020 from the digital documentation system. All patients treated in the University Center for tumor patients (UCT) are followed up after discharge and the time of death is recorded. The records of the patients were screened selecting patients with a primary tumor or brain metastasis who died during hospital stay or less than seven days after discharge. Patients with infratentorial tumor manifestations alone may experience epileptic seizures due to increased intracerebral pressure. However, in the absence of an supratentorial epileptogenic lesion, we excluded these patients from the study. One excluded patient died of a cerebral hemorrhage as a complication of a brain biopsy. A high percentage (72.3%) of those patients received a routine EEG within 45 days prior to death (despite an often palliative setting) due to the special setting of an interdisciplinary ward specialized in neuro-oncology and epileptology. We excluded patients who did not receive an EEG within 45 days prior to their death from the study (Fig. 1). EEG was omited due to several reasons, including the request of no further diagnostics by the patient or a too rapid death after admission.

### Clinical assessment

Clinical signs of seizures were defined in accordance with the International League Against Epilepsy (ILAE) with the difference that solely reduced level of awareness was not classified as a reliable sign of epilepsy as differentiation between seizures/status epilepticus and encephalopathy is clinically not possible in end-of-life patients [14]. Clinical signs therefore included aura symptoms, motor symptoms as well as a gaze deviation. A convulsive status epilepticus is defined as a focal seizure with or without loss of awareness > 10 min or a bilateral tonic-clonic seizure > 5 min by the ILAE [15]. The loss of awareness of patients was separately recorded. For patients who received an EEG, the loss of awareness on the days of the EEG was documented.

### Neuroradiological findings

MRI images of the brain acquired within 3 month prior to the death were used for tumor staging. All MRIs were assessed by a board certified neuroradiologist as well as by at least one consultant of the neuro-oncological team. The evaluation was conducted by the means of the criteria defined by the Response Assessment in Neuro-Oncology (RANO) groups. Here, decisions were solely based on MRI findings excluding the criteria of glucocorticoid usage. According to RANO criteria, the state of the disease is defined as progressive disease (PD), partial or complete response (PR or CR) and stable disease (SD) [16–18]. A further category of initial diagnosis (ID) was added for this analysis.

### Neurophysiological findings

Electroencephalograms (EEGs) were analyzed by two board-certified neurophysiologists. In case of incoherence in the findings between independent readers, the EEGs were reviewed again, and a consensus was reached. The EEGs were scored utilizing the revised 2017 glossary of terms [19] and the 2021 American Clinical Neurophysiology Society’s Standardized Critical Care EEG Terminology [20]. The diagnosis of a status epilepticus was made in coherence to the Salzburg criteria [21].

### Statistics

As we present descriptive data, no statistical analysis has been performed.

## Results

### Clinical background of the cohort

In summary, 68 patients were included into the retrospective study of whom 58.8% (n = 40) had the diagnosis of a primary brain tumor and 41.2% (n = 28) suffered of brain metastasis or a meningiosis carcinomatosa/ lymphomatosa. (Table 1). Noteable, 58.8% of those patients were diagnosed with a structural epilepsy prior to the admission.

**Table 1:** Overview of the cohort

Number of patients (n)		68
Average age (years)		59.1 (standard deviation 12.5)
Type of brain tumor (patients)		
Primary brain tumor		40 (58.8%)
	Glioblastoma	31 (45.6%)
	Others	9 (13.2%)
Secondary brain tumor		28 (41.2%)
	Malignant melanoma	7 (10.3%)
	Non-small cell lung cancer	7 (10.3%)
	Renal carcinoma	1 (1.5%)
	Rectum carcinoma	1 (1.5%)
	Mamma carcinoma	4 (5.9%)
	Meningiosis lymphomatosa	3 (4.4%)
	Esophagus carcinoma	2 (2.9%)
	Merkelcell carcinoma	1 (1.5%)
	No histology available	2 (2.9%)
Radiotherapy within 6 month prior to death (patients)		21 (30.9%)
Active tumor therapy (patients)		34 (50.0%)
Tumor progression based on MRI within the last 3 month (patients)		
	Progressive disease	40 (58.8%)
	Initial diagnosis	17 (25.0%)
	Stable disease	6 (8.8%)
	Partial/complete response	0 (0%)
	No recent MRI available	5 (7.4%)
Known structural epilepy on admission (patients)		40 (58.8%)

### Predominant cause for admission

Patients presented on an average 16.8 days prior to their death (standard deviation 12.2 days, range 1–50 days) and were admitted for one or two of the following reasons. Besides the patients presenting with neurological deficits like paresis or aphasia (26.5%), seizures (32.4%) or status epilepticus (7.4%) were the most common causes for admission. Other causes included delirium and psychosis (13.2%), signs of increased intracranial pressure like headache, nausea and vomiting (10.3%), a decreased general condition (7.4%) and signs of an infection (2.9%).

### Incidence of epileptic seizures determined by clinical observations and routine EEG

**Fig. 2:**
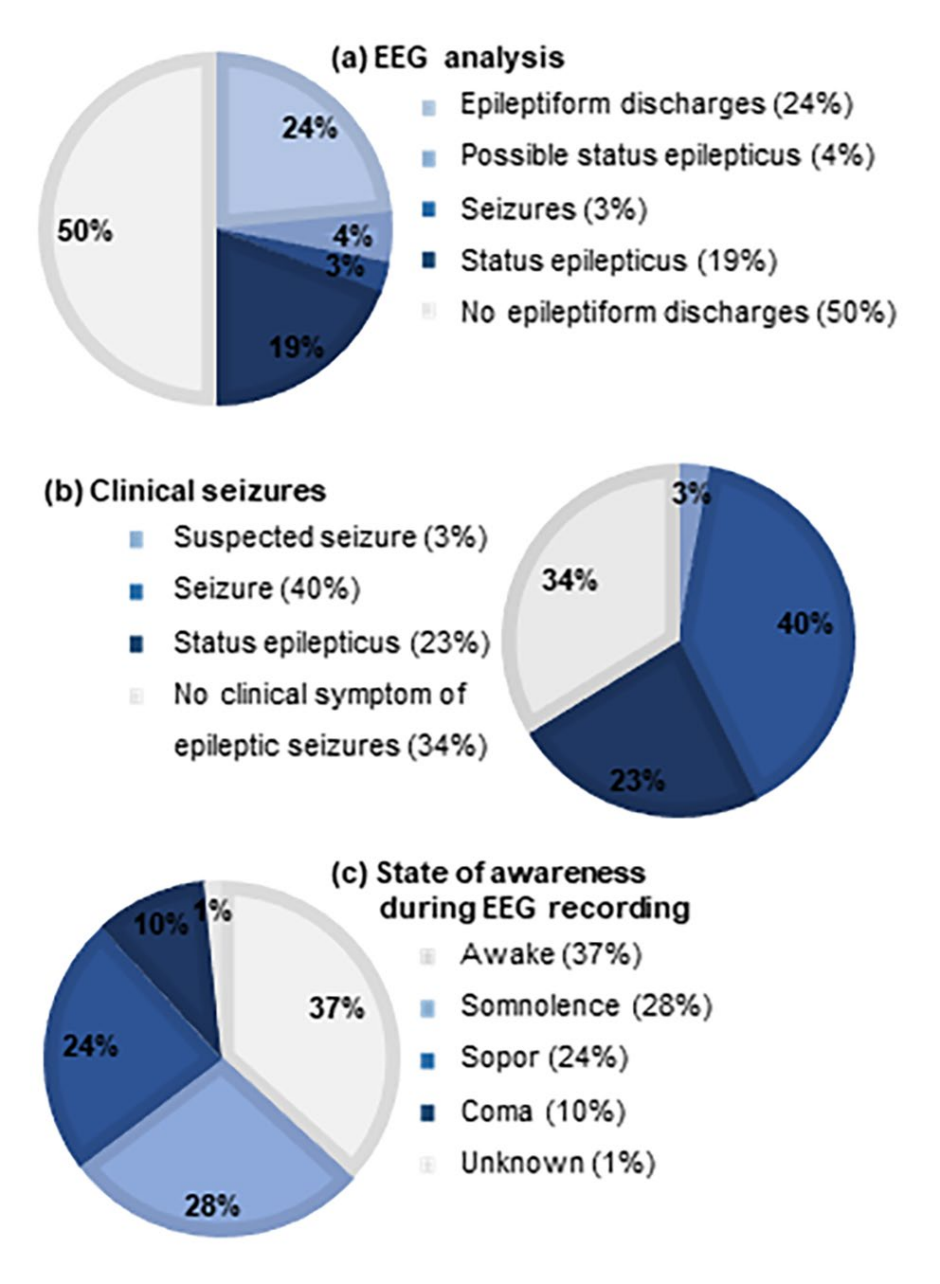
EEG and clinical observations within 45 days prior to death (n = 68). Incidence of seizures determined by EEG analysis (1) and clinical observation (2) as well as the state of awareness of patients during the EEG recording (3)

To estimate the incidence of seizures during the final 45 days of the patient’s life, we analyzed the data for (1) interictal epileptiform discharges/seizure patterns/ status epilepticus on the most pathological routine EEG written on average 11.2 days (standard deviation 10.3 days, range 1–43 days) before death, (2) clinical symptoms of seizures (other than a reduced state of awareness) and (3) episodes of reduced level of awareness which lead to the indication to perform an EEG (Fig. 2).

1. In total 34 patients (50.0%) presented with interictal epileptiform discharges, seizures or status epilepticus patterns in the EEG: The EEG of 16 patients (23.5%) showed interictal epileptiform discharges, two patients presented electrophysiological seizures, 13 patients (19.1%) had a status epilepticus (NCSE n = 11, convulsive SE n = 2) and three patients suffered a possible NCSE according to the Salzburg criteria.
2. According to the documentation, 45 patients (66.2%) presented clinical symptoms of seizures with an average of 11.6 days prior to their death (standard deviation 10.0 days, range 0–44 days). Of those, 27 patients (39.7%) suffered of seizures, 16 patients (23.5%) developed a convulsive status epilepticus (two patients) or status epilepticus with minor motor symptoms or eye deviation (14 patients). Two further patients suffered of a suspected seizure presenting with ictal stigmata like enuresis, tongue bite or distinct myalgia.
3. The state of awareness of the patients at the time the EEG was recorded was reduced in 42 (61.8%) of all patients. Of those patients, 15 (22.1%) presented with seizures or an (possible) status epilepticus in the routine EEG. Another 12 patients (17.6%) showed interictal epileptiform discharges while 15 patients (22.1%) remained without a sign of epilepsy in the EEG of those seven patients (10.3%) did not suffer a clinical seizure either.

**Fig. 3:**
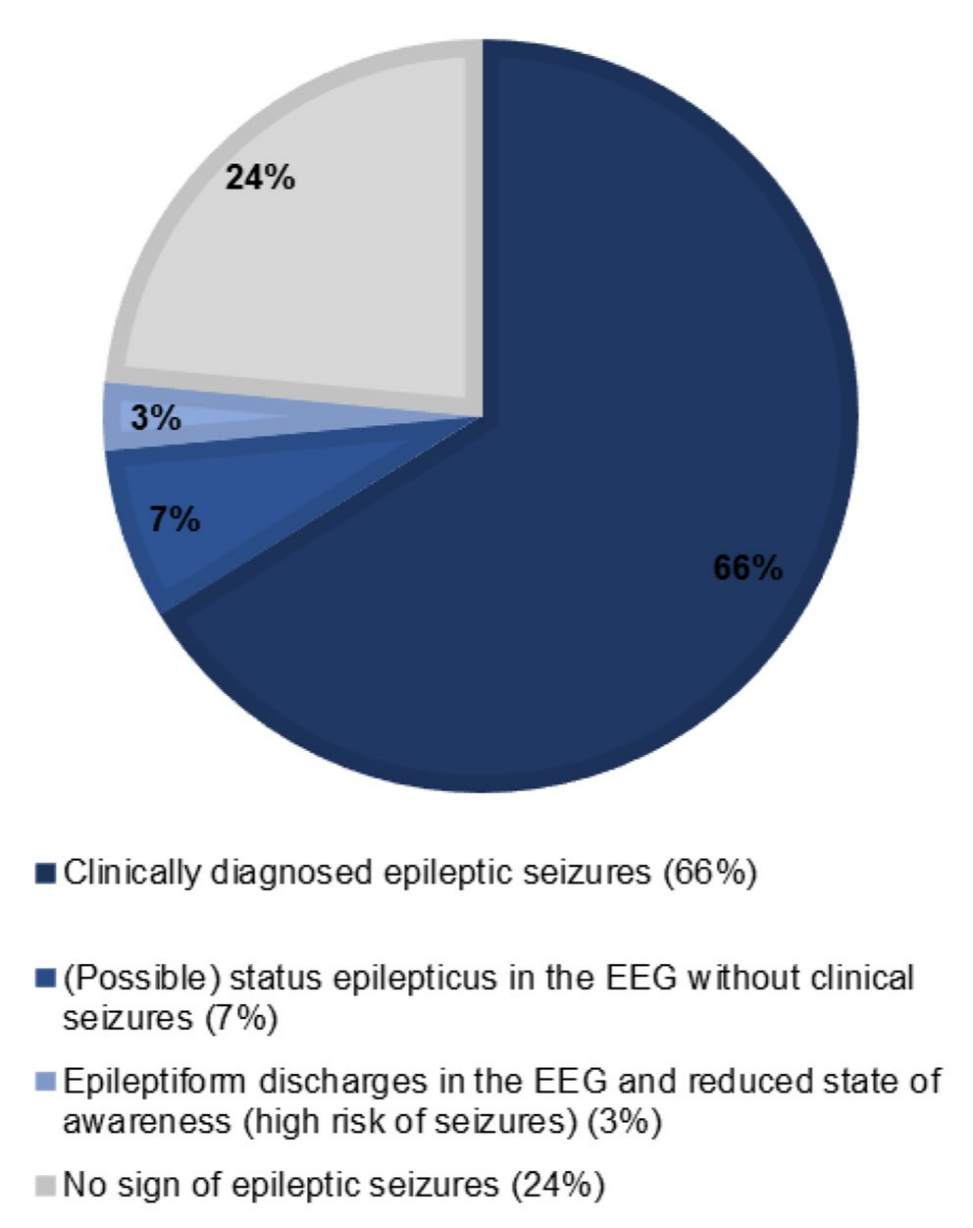
Summary clinical and EEG findings within 45 days prior to death. Epileptic seizures within 45 days prior to death in brain tumor patients combining clinical and electrophysiological findings

To assess whether the electroencephalographic findings are of clinical relevance, we matched the EEG with clinical symptoms of seizures. In summary, 50 patients (73.5%) had either a clinical symptom (other than reduced state of awareness) of epilepsy (45 patients) or presented with a (possible) NCSE in the EEG (five patients) and therefore suffered of seizures/status epilepticus. Another two patients (2.9%) presented only with epileptiform discharges (Fig. 3). All the seven patients without clinical manifestation of seizures suffered of a reduced level of awareness.

When screening the basic demographics including age at the time of death, tumor type, tumor progression and status of therapy of the two cohorts of patients with seizures (n = 50) with patients without seizures (n = 18), no relevant difference could be detected. Due to small numbers of patients, we refrained from depicting the data.

Another nine patients did not suffer any seizures and did not present with any epileptic discharges in the EEG prior to their death but they were previously diagnosed with structural epilepsy and received antiseizure medication. Adding those patients, in total 59 patients (86.8%) had a previously made diagnosis of a structural epilepsy on admission and/or suffered of seizures/ status epilepticus (clinically or on EEG) within 45 days prior to death.

### Patients with or without EEG within 45 days prior to death

To address the bias of the study cohort due to the indication of an EEG, we ran a sub-analysis combining the initially excluded cohort of patients, which did not receive an EEG prior to death (n = 26, Fig. 1) and the examined cohort, which received an EEG within 45 days prior to death (n = 68). We found that 53 of the 94 patients (56.4%) suffered of clinically observed epileptic seizures in the 45 days prior to their death. 52 of 94 patients (55.3%) were diagnosed with a structural epilepsy before they were admitted to the ward. Of those, 25 patients (26.5%) did not suffer seizures within 45 days prior to death. In summary, 78 patients (82.9%) had a previously made diagnosis of a structural epilepsy on admission and/or suffered of clinical epileptic seizures/ status epilepticus within 45 days prior to death.

### Analysis of EEG in the seven days prior to death

For a more focused analysis of the EEG prior to the death, we went on to analyze the EEG of patients which were conducted within seven days prior to their death. From our cohort, 41 patients met the criteria.

Here, 20 patients (48.8%) suffered of a primary brain tumor with 13 patients having the diagnosis of a glioblastoma and 21 patients (51.2%) of a secondary brain tumor. On admission, 24 patients (59%) suffered of a known structural epilepsy. A quarter received radiotherapy within six month prior to their death and 24 patients (59%) received active tumor therapy on admission. Of those patients, 23 patients (56%) suffered of a progressive disease on MRI within three month before and 12 patients (29%) received the initial diagnosis during the admission. Only four patients (10%) had a stable disease on last MRI of the brain.

On the EEG, 15 patients (36.6%) presented epileptiform discharges. Of those, four patients suffered of a status epilepticus, two patients of a possible status epilepticus, one patient of intermittent seizures and eight patients of interictal epilepsy related activity. All of these patients suffered of a reduced level of awareness. Clinically, nine of the 41 patients (22.0%) suffered of clinical signs of seizures (other than a reduced state of awareness) and another eight patients (19.5%) of those of a status epilepticus.

In summary, 21 out of 41 patients (51.2%) had either a clinical symptom (other than reduced state of awareness) of epilepsy or presented with a (possible) NCSE status epilepticus in the EEG (5 patients) and therefore suffered of seizures/status epilepticus. Another seven patients were at high risk for seizures due to epileptiform discharges in the EEG and reduced level of awareness.

### Causes of death

An estimation of the cause of death in patients with a terminal cancer diagnosis is difficult to obtain. The limiting factor is a lack of diagnostics, which are applied towards the end of life. We therefore relied on the final documentation in the medical charts (Table 2). Here, 14 patients (20.6%) were diagnosed to have died as consequence of a status epilepticus/ possible status epilepticus. For most neuro-oncological patients with a limited prognosis, a palliative setting is discussed and decided either according to the will of the patient (phrased by the family) or by his/her own decision. Therefore, 56 of the 68 patients died within a palliative setting including withdrawal of life-prolonging treatment (e.g., antibiotic treatment and artificial nutrition).

**Table 2:** Assumed cause of death*

Status epilepticus/ Estimated status epilepticus	14
Increased intracranial pressure	13
Hemorrhage originating from the brain tumor	4
Pneumonia	22
Urinary tract infection	3
Infect of unknown origin	5
Mulitorgan failure	2
Heart attack/Cardiac shock	4
Unknown	21

*One to two causes of death were stated per patient

## Discussion

In this heterogeneous cohort of supratentorial brain tumor patients, the prevalence of structural epilepsy combined with seizures at the end of life was 86.8% and thus higher than previously reported. The higher detection rate at our center can be attributed to two predominant reasons: Firstly, the wide use of EEG in our center allows a distinction between status epilepticus/ seizures and encephalopathy / hypoactive delirium in patients with a reduced state of awareness. The addition of EEG increased the rate of recognized seizures at the end of life (last 45 days) from 66.2% (clinically diagnosed) to 73.5% (combined with EEG findings). Secondly, the staff is neurologically trained and experienced and the presence of an epilepsy center with accordingly trained neurologists at our site leads to greater attention towards the identification of seizures. Therefore, these numbers of 66.2% of the patients suffering of clinically detected seizures (cohort receiving an EEG within 45 days prior to death, n = 68) exceed the previous numbers reporting an onset of seizures between 6 and 56% [4].

While seizures leading to the initial diagnosis of glioblastoma may be associated with an improved outcome of patients due to an earlier diagnosis, seizures and especially status epilepticus in the course of the disease are accompanied with a decreased overall survival in patients with malignant brain tumors [22]. Although immediate mortality after status epilepticus in brain tumor patients is lower (9%) compared to the overall status epilepticus cohort [22], patients with brain tumors have an increased short term (within 30 days) mortality (17.2% in tumor related status epilepticus versus 11.2% in non-tumor related status epilepticus) [1]. Tumor related status epilepticus/ seizures in the course of the disease are often associated with tumor progression in 49%, thus likely being an epiphenomenon of tumor progression [22]. While an aggressive treatment of brain tumor patients with status epilepticus might be indicated to decrease mortality, this might differ in a palliative setting and needs to be evaluated based on the individual scenario.

Consequent antiseizure treatment results in a reduction of distress of the caregivers as well as the prevention of repetitive presentations at the hospital [23, 24]. Treatment at that time is primarily with benzodiazepines and intravenous and oral antiseizure medications; escalation therapy with general anesthesia with ventilation is not indicated [25, 26]. Adequate treatment and therefore prevention of NCSE can lead to an improved vigilance of the patients. On the other hand, tumor related epilepsy is often resistant to medical management and provocation factors lead to a relative inefficiency of antiseizure treatment at the end of life and therefore polytherapy [27]. Here, the side effects of this antiseizure polytherapy weight more severe on the quality of life in tumor patients as the seizure frequency [28]. Furthermore, antiseizure medication may lead to an unnecessary and unwanted prolongation of patient’s life [12].

In summary, more patients than previously reported suffer of seizures at the end of life with sometimes subtle or no specific clinical features. Therefore, patients and caregivers must be instructed about the high incidence of seizures and status epilepticus during that stage of disease. The decisions on escalated antiseizure treatment cannot be based on the sole presence of seizures but must be estimated individually according to the burden of the seizures for the patients and their surroundings, the side effect of antiseizure medication as well as on patients’ wishes in order to preserve the best possible quality of life.

## Limitations

While being a strength of the study, the setting in a tertiary center specialized in neuro-oncology and epileptology also represent a limitation. Firstly, patients with brain metastasis at the end of life may be underrepresented in a neuro-oncological center as they only present when they suffer from neurological symptoms. Secondly, admission on the ward pose a bias regarding patient selection (27 of the 68 examined patients were admitted due to seizures/status epilepticus). Another limitation represents that the exclusion of patients who did not receive a routine EEG in the 45 days before death may create a bias towards an increased incidence of seizures.

## Data Availability

The datasets generated during and/or analysed during the current study are available from the corresponding author on reasonable request.
